# Philadelphia chromosome-positive B-cell acute lymphoblastic leukemia with e1a3 *BCR-ABL1* transcript in a Nigerian with sickle cell anemia: a case report

**DOI:** 10.1186/s13256-021-03060-5

**Published:** 2021-10-08

**Authors:** Ibrahim O. Ahmed, Lauretta O. Ochogwu, Temilola O. Owojuyigbe, Norah O. Akinola, Muheez A. Durosinmi

**Affiliations:** 1 Department of Haematology and Blood Transfusion, Obafemi Awolowo University Teaching Hospitals Complex (OAUTHC), Ile-Ife, Nigeria; 2 Department of Haematology and Immunology, Obafemi Awolowo University (OAU), Ile-Ife, Nigeria

**Keywords:** Acute lymphoblastic leukemia, Sickle cell anemia, *BCR-ABL1*, e1a3, Case report

## Abstract

**Background:**

The occurrence of acute leukemia in patients with sickle cell anemia is uncommon. The Philadelphia chromosome is the hallmark of chronic myeloid leukemia. However, it may also be associated with acute lymphoblastic leukemia and acute myeloblastic leukemia. The common *BCR-ABL1* transcripts seen in acute lymphoblastic leukemia are e1a2, e13a2, and e14a2, while other transcripts such as e1a3, e13a3, and e6a2 occur rarely. This report describes the presentation, management, and outcome of the occurrence of B-cell acute lymphoblastic leukemia with the rare e1a3 *BCR-ABL1* transcript in a patient with sickle cell anemia.

**Case presentation:**

A 19-year-old male Nigerian, a known sickle cell anemia patient was admitted on account of severe vaso-occlusive crisis. Examination revealed fever, palor, and jaundice. Full blood count showed anemia and leukocytosis. Peripheral blood and bone marrow smears revealed numerous large and small lymphoblasts in keeping with the L2 subtype of acute lymphoblastic leukemia based on the French–American–British classification. Further evaluation was in keeping with a diagnosis of *BCR-ABL1*-positive mature B-cell acute lymphoblastic leukemia associated with the rare e1a3 transcript. He was commenced simultaneously on induction chemotherapy and Imatinib while being prepared for allogeneic stem cell transplantation. However, he died  six  months after diagnosis from meningoencephalitis.

**Conclusion:**

The occurrence of acute lymphoblastic leukemia with a rare *BCR-ABL1* e1a3 transcript in association with sickle cell anemia is uncommon and associated with poor prognosis.

## Introduction

Sickle cell anemia (SCA) is the most prevalent hemoglobinopathy worldwide and occurs due to the homozygous inheritance of hemoglobin S (HbS) [1]. Hemoglobin S is an abnormal form of adult hemoglobin (HbA) that is produced due to a point mutation in the sixth codon of the beta globin gene (GAG to GTG) resulting in the replacement of the hydrophilic amino acid glutamate by the hydrophobic amino acid valine in the sixth position of the Hb beta chain. The abnormal HbS polymerizes when deoxygenated and induces sickling of red blood cells (RBCs). The clinical features include chronic hemolysis, anemia, and periodic episodes of acute pain termed vaso-occlusive crisis (VOC) [2]. The occurrence of acute leukemia in individuals with SCA is rare [2, 3]. This has been attributed to the reduced life expectancy associated with SCA. The Philadelphia chromosome (Ph) is the shortened chromosome 22 that results from the reciprocal translocation between the long arms of chromosomes 9 and 22, that is, (t9;22)(q34:q11) with the formation of a novel fusion gene *BCR-ABL1* that drives oncogenesis due to increased tyrosine kinase activity [4]. This acquired gene rearrangement is most classically associated with CML, but it could also be found in ALL and AML [4, 5]. There are many variants of the *BCR-ABL1* fusion gene depending on the site of breakpoint in the *BCR* gene [5]. The most common *BCR-ABL1* fusion transcript seen in about 70% of Ph-positive ALL is the p190 variant, which results from the rearrangement between exon 1 of *BCR* and exon 2 of ABL1, that is, e1a2. About 30% of cases of Ph-positive ALL are associated with the p210 variant [that has two forms, namely e13a2 (or b2a2) and e14a2 (or b3a2)] [6]. Baccaranni *et al*., however, reported the occurrence of e13a2 transcript in 58.5% of 497 *BCR-ABL1* positive ALL patients [7]. Atypical variants of the *BCR-ABL1* gene such as e1a3, e13a3, and e6a2 occur rarely [6, 8]. The presence of *BCR-ABL1* transcript in ALL is associated with a relatively poor prognosis, necessitating the addition of tyrosine kinase inhibitor (TKI) into the treatment regimen [7].

Philadelphia-chromosome positive (Ph+) acute lymphoblastic leukemia is a distinct entity that is characterized by specific genomic alterations, low sensitivity to chemotherapy, unstable responsiveness to tyrosine kinase inhibitors (TKIs), and a poor prognosis [8, 9].

We report a rare case of Ph-positive B-cell ALL with e1a3 *BCR-ABL1* transcript in a 19-year-old Nigerian with SCA.

## Case presentation

A 19-year-old Nigerian, known SCA patient who was diagnosed in childhood but had been receiving care in a secondary health facility presented to the emergency room in May 2020 with generalized body pains, fever, weakness, and palpitations of four days duration. There was no history of passage of dark-colored urine, thus excluding hemolysis. The patient had never used hydroxycarbamide nor any other disease-modifying agent. He was diagnosed to have SCA at the age of 1 year during a febrile illness when a routine Hb electrophoresis was done. In early childhood, he had frequent episodes of VOC that required hospitalization (more than four times annually) and required numerous blood transfusions (more than three times in a year). However, in his teenage years, VOCs were said to have become less frequent (once or twice in a year) and less intense with significant reduction in hospital admission rate (once or twice in a year) and blood transfusion requirements (less than once in a year). The last episode of crises that required hospitalization (at a private hospital in Port-Harcourt, Nigeria) occurred a year prior to index presentation. The last blood transfusion was at age 15 years. He did not attend any clinic for follow-up, but presented mainly at the emergency room during crises when home treatment was not effective. He did not have splenectomy as this is not routinely done in Nigeria for patients with SCA. He was not compliant with the prescribed daily medications, which included proguanil, folic acid, vitamin B-complex, and vitamin C. He was the last of three children in a monogamous family, in which both parents and his siblings had Hb AS phenotype.

**Fig. 1:**
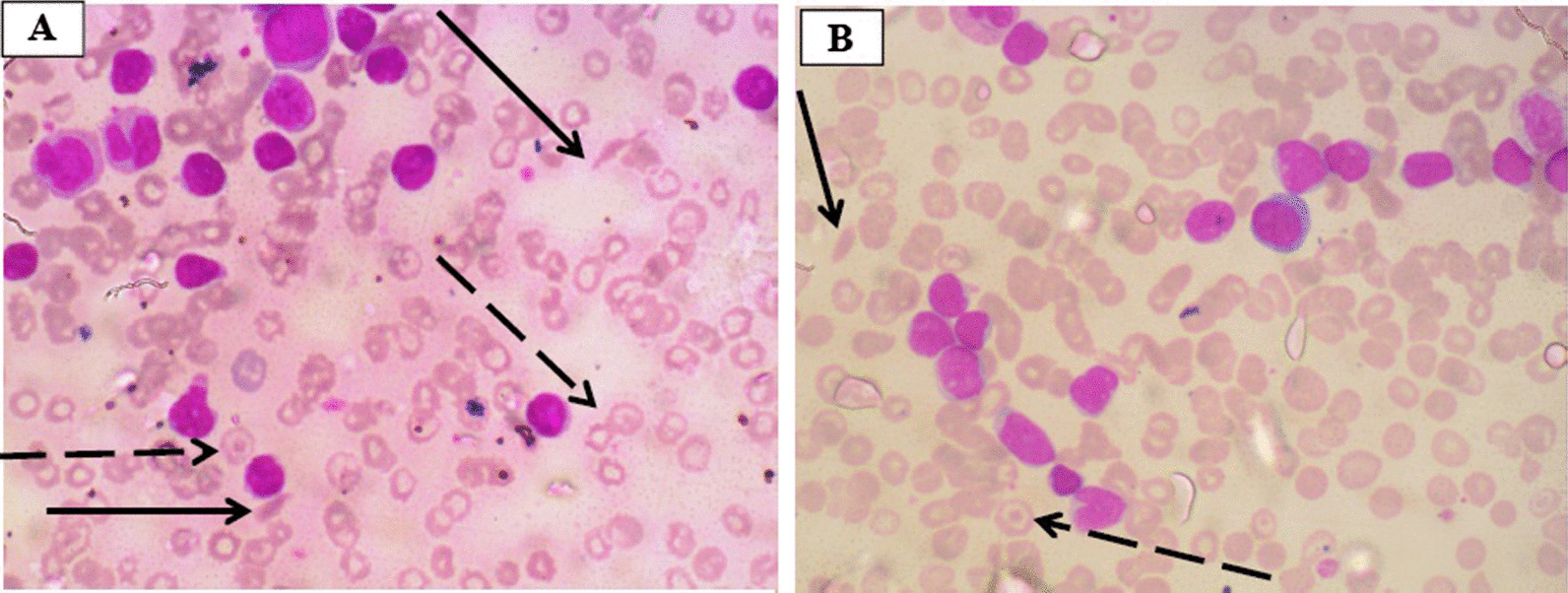
Peripheral blood film of the patient. Different views of the peripheral blood film of the patient showing numerous heterogeneous lymphoblasts, sickle cells (solid arrows), and target cells (dashed arrows)

**Fig. 2:**
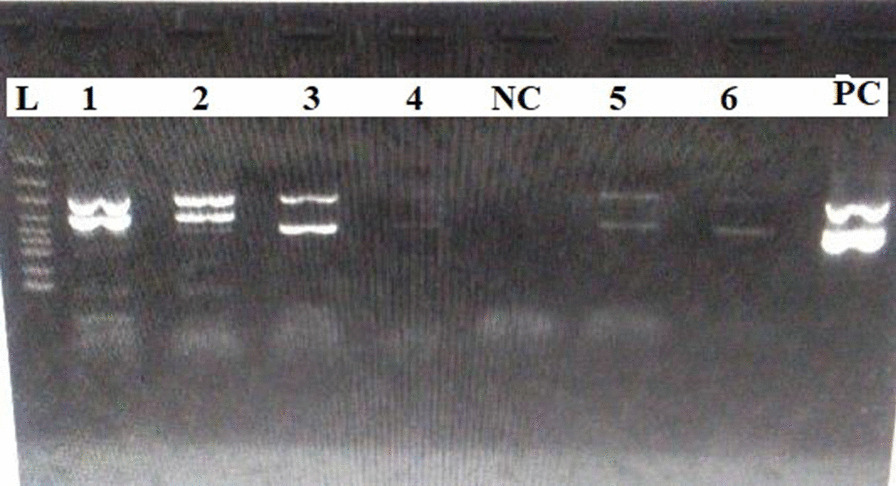
Polymerase chain reaction analysis of *BCR-ABL1* transcripts. Lane L: DNA ladder, Lane 1: e1a3 (index patient), Lane 2: e13a2 + e14a2, Lane 3: e14a2, Lane 4: e14a2 (low value), NC: negative control, Lane 5: e14a2, Lane 6: e14a2, PC: positive control

Examination revealed a young man in painful distress with pyrexia (temperature 38.3 °C), palor, slight jaundice, no significant peripheral lymphadenopathy, and no pedal edema. Respiratory rate was 24 breaths/minute. Pulse was 120 beats/minute, regular, but of small volume. Blood pressure was $\frac{120}{70}$ mmHg, while the apex beat was palpable at the fifth left intercostal space, mid-axillary line. The first (S_1_) and second (S_2_) heart sounds were heard. There was no abdominal tenderness, but the liver was palpably enlarged, 4 cm below the right costal margin and had a span of 18 cm. The spleen was not palpable, and the kidneys not ballotable. He had no neurological abnormality. Baseline full blood count (FBC) showed severe anemia (hemoglobin concentration 4.6 g/dl), leukocytosis (WBC 44.3 × 10^9^/L) and platelet count of 335.0 × 10^9^/L. Blood film microscopy for malaria showed trophozoites of *Plasmodium falciparum*. Hb electrophoresis on agarose gel with densitometric quantification (Hellabio, Thessaloniki, Greece) done at presentation revealed HbS 93.6%, HbF 2.9%, HbA_2_ 3.5%. Steady-state hematocrit was unknown. An initial diagnosis of VOC with severe anemia was made. He was treated with artesunate-based antimalarial and transfused with packed cells. However, a review of the peripheral blood film showed sickled and target cells in addition to leukocytosis that consisted of heterogeneous lymphoblasts (81%), myelocytes (2%), neutrophils (10%), and lymphocytes (7%) (Fig. 1). Urine analysis was normal. Abdominal ultrasound scan showed hepatomegaly and autosplenectomy. Electrocardiogram (ECG) showed left ventricular hypertrophy (LVH), while echocardiogram (ECHO) showed LVH with normal systolic and diastolic function. Serology tests for human immunodeficiency virus (HIV), hepatitis B virus, and hepatitis C virus were negative. Bone marrow aspiration (BMA) revealed a hypercellular marrow with numerous heterogeneous lymphoblasts constituting about 60% of marrow nucleated cells. Flow cytometry of the peripheral blood revealed blasts that were CD20+, CD3−, CD13−, and CD33−. Multiplex reverse transcriptase polymerase chain reaction (RT-PCR) was done for *BCR-ABL1* analysis (Seeplex Leukemia BCR-ABL kit, Seegene, Seoul, Korea) and showed the presence of e1a3 transcript (Fig. 2). A diagnosis of B-lineage *BCR-ABL1* positive ALL in a known patient with SCA was made. Lymphoblasts were not visualized on cytospin analysis of CSF, thus excluding CNS involvement. However, he was classified as being at high risk for meningeal relapse on the basis of high WBC count (WBC >30.0 × 10^9^/L), mature B-cell phenotype and Ph expression.He was commenced on the modified MCP 841 regimen (Table 1) [10, 11]. This protocol included four induction cycles (1A, 2A, 2B, 1B); one cycle of consolidation and six cycles of maintenance therapy. Induction 1A involved the administration of prednisolone, vincristine, l-asparaginase, and daunorubicin. He was also given three-drug intrathecal therapy (IT) [methotrexate, hydrocortisone, and cytarabine, that is, triple IT] by aseptic technique for CNS prophylaxis as per protocol for patients at high risk for CNS relapse during induction and consolidation. Cytospin analysis of the CSF was performed routinely prior to each IT, and no lymphoblasts were seen. In addition, Imatinib mesylate (Novartis Pharmaceuticals, Basel, Switzerland) 600 mg was given simultaneously, courtesy of The Max Foundation (Max Access Solutions^©^, The Max Foundation, Seattle, Washington, USA). Packed red cell and platelet transfusions were given as necessary. Induction 1A was complicated by neutropenic fever, sepsis and pityriasis versicolor for which he received granulocyte colony stimulating factor (G-CSF), antibiotics (ciprofloxacin, metronidazole), acyclovir, topical clotrimazole and fluconazole. Co-trimoxazole was also given as prophylaxis against *Pneumocystis jirovecii*. Samples were taken from the two siblings and the parents for human leukocyte antigen (HLA) typing in preparation for allogeneic hemopoietic stem cell transplantation (Allo-HSCT). He achieved remission after induction 1A as confirmed by BMA on day 30. The *BCR-ABL1* analysis and flow cytometry could not be repeated due to financial constraints. Induction 2A and 2B was complicated by the development of multiple subcutaneous abscesses. Microbiological culture yielded methicillin-resistant *Staphylococcal aureus*. He was treated with meropenem and linezolid. He continued chemotherapy until the consolidation phase when, exactly  six months from diagnosis, he developed high-grade fever, headache and vomiting. On admission at the emergency department, he was restless with altered consciousness, fever (temperature 39.4 °C), and palor. The Glasgow Coma Scale (GCS) score was 9 out of 15 based on eye opening to pain, utterance of incomprehensible sounds, and localization of pain. The pupils were both 3 mm in diameter with normal light reactivity. He had neck stiffness and generalized hypertonia, with positive Kernig and Brudzinski signs. Pulse rate was 122 beats per minute; blood pressure was $\frac{144}{70}$ mmHg. A diagnosis of meningoencephalitis with raised intracranial pressure (ICP) was made. The clinical suspicion of raised ICP was based on headache, vomiting, and increased blood pressure that prevented the collection of CSF for cytology and culture. He was placed on intravenous ceftriaxone 2 g 12-hourly and intravenous dexamethasone 8 mg 8-hourly, but died within a few hours in the intensive care unit (ICU) while arrangements were being made for cranial computed tomography (CT) scan. The relatives declined autopsy.

**Table 1:** Modified MCP 841 Protocol

Phase and duration	Medication	Dosage Route and schedule
Induction 1A 29 days	1. Pre-induction prednisolone	60 mg/m^2^ PO day 1–7
	2. Prednisolone	40 g/m^2^ PO day 8–28
	3. Vincristine	1.4 mg/m^2^ IV day 8, 15, 22, 29
	4. Daunorubicin	20 mg/m^2^ IV day 8, 15, 29
	5. l-Asparaginase	6000 U/m^2^ SC day 8, 10, 12, 14, 16, 18, 20, 22, 24, 26
	6. CNS prophylaxis: *Triple prophylaxis	
	Methotrexate	12 mg; IT day 8, 15, 22, 29
	Cytarabine	30 mg; IT day 8, 15, 22, 29
	Hydrocortisone	20 mg; IT day 8, 15, 22, 29
Induction 2A 28 days	Cyclophosphamide	750 mg/m^2^; IV day 1, 15
	6-Mercaptopurine	60 mg/m^2^ ; PO day 1–28
	Cytarabine	75 mg/m^2^; IV day 1–4, 8–11, 15–18, 22–25
	CNS prophylaxis as Induction 1A	IT day 8, 15, 22, 29
Induction 2B 56 days	Cyclophosphamide 6-Mercaptopurine	750 mg/m^2^; IV day 1, 15 60 mg/m^2^; PO day 1–56
	Methotrexate	**Low risk**: 20 mg/m^2^; IV day 1, 11, 21, 31, 41 **High risk**: Start at 100 mg/m^2^ and increase by 50 mg/m2 up to 5 doses
	CNS prophylaxis as in Induction 1A	IT day 8, 15, 22, 29
Induction 1B 28 days	Repeat Induction 1A	As in Induction 1A
Consolidation 28 days	Cytarabine Vincristine Cyclophosphamide Daunorubicin 6-Mercaptopurine	100 mg/m^2^; SC 12-hourly day 1–3, 15–17 1.4 mg/m^2^; IV day 1, 15 750 mg/m^2^; IV day 1 20 mg/m^2^; IV day 15 75 mg/m^2^; PO day 1–7, 15–21
	CNS prophylaxis:- Drugs as in Induction 1A	IT day 1
Maintenance 8 cycles for B-ALL (12 weeks per cycle) 96 weeks	Prednisolone Vincristine Daunorubicin l-Asparaginase 6-Mercaptopurine Methotrexate	40 g/m^2^; PO day 1–7 1.4 mg/m^2^; IV day 1 20 mg/m^2^; IV day 1 6000 U/m^2^; SC day 1, 3, 5, 7 75 mg/m^2^; PO daily × 12 doses 15 mg/m^2^; PO weekly
	CNS prophylaxis:- Methotrexate	12 mg; IT day 1 of each cycle

## Discussion

The life expectancy of patients with SCA has improved in recent years owing to advances in management that may be associated with the many cases of malignancies now being reported in these patients. In Nigeria, 8.6% of cases of acute leukemia were associated with sickle cell disease, among which there was only one case of ALL [2]. Brunson *et al*. found an increased risk of leukemia in patients with SCD when compared with the general population [12]. The factors associated with this increased risk include chronic inflammation, increased iron levels from frequent transfusions, increased risk of infections, increased erythroid proliferation, and increased bone marrow turnover, which form the pathophysiologic mechanisms of the clinical manifestations of SCD [12]. The index patient presented with typical clinical features of VOC and severe anemia, which occur commonly in patients with SCA. The detection of lymphoblasts in the peripheral blood smear provided the clue to the diagnosis of ALL, which was confirmed by further investigations such as BMA, *BCR-ABL1* analysis by PCR, and the immunophenotype. The clinical phenotype of SCA in the patient had been severe with frequent episodes of VOCs and blood transfusions, which were said to have become ameliorated in his teenage years. The ECG and echocardiographic findings of LVH were consistent with chronic anemia, which also occurs frequently in patients with SCA [13].

The *BCR-ABL1* fusion gene results from the reciprocal translocation between the long arms of chromosomes 9 and 22, that is, t(9;22)(q34;q11) [4]. The *ABL1* tyrosine kinase gene is transferred from its normal position on chromosome 9 to a new position beside the *BCR* gene on chromosome 22, resulting in the generation of a novel, highly oncogenic fusion gene (*BCR-ABL1*) with a very high constitutive tyrosine kinase activity. This *BCR-ABL1* gene is expressed in 95% of patients with CML, 25–30% of adult ALL, and 2–3% of ALL in children [5, 14, 15]. The most common *BCR-ABL1* rearrangement in ALL is the e1a2 transcript with a molecular weight of 190 kDa (that is, p190), but the e13a2 (or b2a2) and e14a2 (or b3a2) transcript variants occur less frequently [16]. Other variants such as the e1a3 as reported in this index case are rarely seen in ALL, resulting from the translocation between exon 1 of *BCR* on chromosome 22 and exon 3 of *ABL1* on chromosome 9 [15]. The exon 2 (a2) region of *ABL1* that encodes a part of the Src homology (SH) 3 domain is missing from the e1a3 *BCR-ABL1* transcript. The role of the SH3 domain in leukemogenesis is controversial. It is a negative regulator of the kinase domain (SH1) of *ABL1* and *BCR-ABL1* [15]. Therefore, the absence or mutation of the SH3 domain may be associated with a more aggressive form of Ph-positive leukemia due to the loss of its autoinhibitory effect on kinase activity. On the other hand, the SH3 domain is also required for STAT5 activation by the BCR–ABL1 protein, which is necessary for the expression of its full leukemogenetic effect. In this instance, mutation of SH3 domain could be associated with a slowly progressive disease. In CML with the e1a3 transcript, the lack of the SH3 domain was associated with inferior response to Imatinib when compared with the major transcripts and rapid progression to blastic crisis [17]. In other CML patients, the e1a3 transcript was associated with better outcomes [14, 16, 18, 19].

The clinical profile of e1a3 is expected to be similar to that of e1a2 because of their identical molecular weight (190kDa). However, in CML, the e1a2 transcript is associated with monocytosis, extramedullary infiltration, and rapid disease progression similar to chronic myelomonocytic leukemia (CMML) [19].

B-cell acute lymphoblastic leukemia (B-ALL) occurs mainly in children and young adults. The disease is characterized by the expression of B-cell antigens CD19, CD20, or CD22. In most cases, the malignant B lymphocytes are immature (precursor B lymphocytes), while mature lymphocytes are found in only 1–2% of cases. This is frequently associated with the expression of various cytogenetic abnormalities that produce fusion genes such as *BCR-ABL1* or *TEL-AML1* as well as abnormalities in signaling pathway genes such as *RAS*, *PAX5*, or *PI3K* [20]. Philadelphia chromosome-positive ALL is characterized by an aggressive clinical course, poor response to conventional chemotherapy, and poor survival rates [21]. The incorporation of TKIs into the treatment of Ph+ALL is associated with better survival [20, 22]. Imatinib is provided in Nigeria free by The Max Foundation since 2003 through its patient assistance program (Max Access Solutions^©^, The Max Foundation, Seattle, Washington, USA). Second-generation TKIs such as Nilotinib or Dasatinib were not available in our center when the patient was managed. Monoclonal antibodies such as rituximab may also be used for therapy [20].

The MCP 841 chemotherapy protocol is a multiagent, multiphase, moderate-intensity regimen that was developed through a collaboration between the Cancer Institute (WIA), Chennai, India and the National Cancer Institute (NCI), Bethesda, USA. It is a standardized treatment protocol that has led to improved survival rates and reduction in treatment-related toxicity among pediatric and adolescent ALL patients treated in resource-poor settings. In the MCP 841 protocol, CNS prophylaxis involves IT alone (in patients younger than 3 years of age) or with cranial irradiation (in patients older than 3 years of age) [10, 11]. Cranial irradiation is effective, but frequently complicated by significant short-term and long-term toxicity, such as, neurocognitive dysfunction, endocrinopathy, secondary neoplasms, and neurotoxicity [23]. Studies have shown that cranial irradiation can be safely omitted from CNS prophylaxis even in high-risk adult or pediatric patients by means of effective systemic chemotherapy and intensive triple intrathecal therapy [24–26]. The protocol modification used in this case involved the replacement of cranial irradiation with the Capizzi escalating-dose systemic methotrexate and triple IT for CNS prophylaxis [26, 27], because there are no facilities for cranial irradiation in our center.

There are relatively few studies on the significance of *BCR-ABL1* transcript types in ALL. Some reports have described the presence of the e1a3 transcript in ALL; however, to the best of our knowledge, this is the first report of such in a patient with SCA [28, 29]. In addition, this is the first report from Nigeria in which cytogenetic and immunophenotype analyses were done in the diagnosis of acute leukemia in any patient with SCD. There is limited utilization of cytogenetics and flow cytometry in the diagnostic workup of acute leukemia patients in Nigeria owing to lack of facilities in most centers [2, 3].

Fujisawa *et al*. reported e1a3 transcript in a 25-year old female with precursor B-ALL who presented with leukocytosis and thrombocytopenia. Similar to this index case, the patient had chemotherapy and Imatinib therapy simultaneously and achieved complete remission after the first induction cycle. However, the patient succumbed to veno-occlusive disease 18 months from the date of diagnosis [30]. Similarly, Langerbeer *et al*. reported this rare transcript in a 62-year-old female with Pre-B ALL who also had concomitant chemotherapy with Imatinib therapy. The patient achieved remission with fludarabine, cytarabine, G-CSF, and idarubicin, but relapsed at 18 months and died at 27 months [28]. Lopez-Andrade *et al*. reported two patients ALL with the e1a3 *BCR-ABL1* ALL transcript. In both patients, the *BCR-ABL1* transcript remained detectable after induction chemotherapy despite the addition of Imatinib. The first patient achieved complete response by morphology and immunophenotype. The second patient did not respond to treatment based on the presence of lymphoblasts in the post-induction BMA and persistence of *BCR-ABL1*; he died after second-line induction due to gastrointestinal bleeding [31].

Burmeister *et al*. of the German Multicenter ALL (GMALL) Study Group detected *BCR-ABL1* transcripts in 34% of 1214 adult B-precursor ALL cases. Eight of these patients had atypical transcripts, five of which were e1a3, two e13a3, and one e6a2 [6].

In all the previously reported cases, the patients died between  nine and 27 months unlike this index patient, who died within  six months of diagnosis. The shorter duration of survival reported in this case might be due to the associated comorbidities, namely the SCA and meningoencephalitis, which complicated the treatment. Infections are commonly implicated as contributory factors to mortality in ALL patients. In the index patient, an increased risk of severe infection could be linked to impaired cellular and humoral immunity from the cumulative effects of SCA, ALL, and cancer chemotherapy [32].

Nigeria is located within the “meningitis belt” in Sub-Saharan Africa, and outbreaks of meningitis caused by *Streptococcus pneumoniae*, *Neisseria meningitidis*, and *Haemophilus influenzae* occur frequently [33]. Methicillin-resistant *Staphylococcal aureus* could also be a cause in the patient considering the history of previous soft-tissue infection caused by the organism in him. In immunocompromised individuals, meningitis could be caused by atypical organisms such as *Listeria monocytogenes*, *Cryptococcus neoformans*, *Acanthamoeba* species, or herpes simplex virus [34]. The other differential diagnoses of meningoencephalitis in the patient include leukemic meningitis, aseptic meningitis, and drug-induced meningitis (from IT therapy) [35].

While numerous variant *BCR-ABL1* transcripts and their responses to a variety of treatment modalities have been documented in CML [15, 17, 36]. There are fewer reports on these rare variants in Ph+ ALL: the largest series examined to date reveals a total incidence of 1.9%, with the e1a3 transcript type being the most frequently detected at about 1.2% [6]. All the patients had induction therapy with or without Imatinib. The patients who were treated with Imatinib had better survival after HSCT and less risk of relapse than those who were not treated with Imatinib. The time to HSCT ranged from 100 to 180 days after diagnosis [6]. The index patient died before HSCT could be done on day 180.

## Conclusion

The occurrence of ALL with the rare e1a3 *BCR-ABL1* transcript in SCA is associated with poor prognosis. There is need to develop standardized treatment protocols to improve survival. Stem cell transplantation should be made available to patients as early as possible to improve survival. Routine examination of the peripheral blood film of patients with SCA presenting with features of crises is recommended. Molecular techniques should be included in the diagnostic workup of patients with acute leukemia.

## Data Availability

All data generated or analyzed in this study are included in this published article.

## Abbreviations

ALL: Acute lymphoblastic leukemia
AML: Acute myeloblastic leukemia
CCR: Complete cytogenetic remission
CD: Cluster of differentiation
CML: Chronic myeloid leukemia
CMML: Chronic myelomonocytic leukemia
CNS: Central nervous system
CSF: Cerebrospinal fluid
ECG: Electrocardiogram
ECHO: Echocardiogram
FAB: French–American–British (classification)
FBC: Full blood count
GCS: Glasgow Coma Scale
G-CSF: Granulocyte colony stimulating factor
GMALL: German Multicenter ALL (study group)
Hb: Hemoglobin
HIV: Human immunodeficiency virus
HLA: Human leukocyte antigen
HSCT: Hemopoietic stem cell transplantation
ICU: Intensive care unit
ICP: Intracranial pressure
IT: Intrathecal therapy
NCI: National Cancer Institute, Bethesda
PCR: Polymerase chain reaction
RBC: Red blood cell
SCA: Sickle cell anemia
SCD: Sickle cell disease
TKI: Tyrosine kinase inhibitor
USA: United States of America
WBC: White blood cell
WIA: Cancer Institute, Chennai, India
